# Laparoscopic sleeve gastrectomy *versus* Roux-en-Y gastric bypass for morbid obesity: a 1:1 matched cohort study in a Chinese population

**DOI:** 10.18632/oncotarget.12536

**Published:** 2016-10-08

**Authors:** Xiao Du, Si-qin Zhang, Hong-xu Zhou, Xue Li, Xiao-juan Zhang, Zong-guang Zhou, Zhong Cheng

**Affiliations:** ^1^ Department of Gastrointestinal Surgery, Laboratory of Bariatric and Metabolic Surgery, West China Hospital, Sichuan University, Chengdu, P.R. China; ^2^ Department of Endocrinology and Metabolism, West China Hospital, Sichuan University, Chengdu, P.R. China

**Keywords:** bariatric surgery, Roux-en-Y gastric bypass, sleeve gastrectomy, morbid obesity, weight loss

## Abstract

**Objectives:**

This 1:1 matched cohort study with 3-year follow-up aimed to compare the safety and efficacy of LSG with LRYGB for morbid obesity patients.

**Methods:**

From 2009 to 2013, patients undergoing LRYGB (*n* = 63) were matched with LSG (*n* = 63) by gender, age, and body mass index (BMI). Major complications, BMI, percentage of excess weight loss (%EWL), and obesity-related comorbidities after 6, 12, 24, and 36 months were compared.

**Results:**

Hospital stay and major complication rates were comparable, but operative time in LSG was significantly shorter (83.2 ± 23.7 *vs*. 108.3 ± 21.3 min). No significant differences in mean %EWL and BMI were observed at 6, 12, 24 months. At 3-year follow-up, mean %EWL in the LRYGB group was significantly higher than in the LSG group (76.5 ± 9.2% vs. 65.7 ± 10.3%) and, consequently, mean BMI was significantly lower in LRYGB (28.2 ± 1.5 *vs*. 30.9 ± 2.4 kg/m2). No significant differences in remission of comorbidities were observed at 1- or 3-year follow-up.

**Conclusions:**

Both LRYGB and LSG were safe and effective bariatric procedures in this Chinese population, but LRYGB seemed to be superior to LSG in terms of mid-term weight loss.

## INTRODUCTION

Obesity has become a worldwide public health problem in recent years. It is estimated that more than 1.9 billion people in the world are overweight or obese.[1] Many developing countries, including China, are facing an epidemic of obesity and the challenges of obesity-related diseases.[2] Although diet control, physical exercise, and medication can induce some amount of weight loss, studies from Western countries have shown that bariatric surgery is the only treatment capable of providing substantial and sustainable weight loss in morbid obesity. However, in the Orient, obese people often carry severe intra-abdominal fat accumulation with only moderately elevated body mass index (BMI). The benefit of bariatric surgery in Eastern populations is still uncertain, especially in China, where this technique has been in use for no more than 20 years.

A wide range of procedures are available in the ever-growing field of bariatric surgery. They can be divided into 3 types by the mechanism of action: restriction, mal-absorption or a combination of both. There are still no established criteria to aid selection of patients for a specific procedure. Laparoscopic Roux-en-Y gastric bypass (LRYGB), considered as the gold standard procedure, always presents its substantial, long-term effects on both weight loss and resolution of comorbidities.[3, 4] However, it is technically highly demanding, requiring a long learning curve and advanced surgical skills; serious complications are also possible. In contrast, laparoscopic sleeve gastrectomy (LSG) is a comparatively ease and safe procedure as no anastomosis or foreign body implantation is required. Researchers comparing the two procedures have reported conflicting results, and there are few studies from the Orient. Which procedure is more suitable for Chinese patients is still under investigation.

Here we report the early and mid-term outcomes of LRYGB and LSG procedures in our institute. The aim of this retrospective 1:1 matched cohort study was to compare efficacy and safety between LRYGB and LSG in morbidly obese Chinese patients.

## RESULTS

A total of 126 patients were enrolled into this study, of whom 63 underwent LRYGB and 63 underwent LSG. Both groups were comparable in terms of sex, age, BMI, waist circumference and comorbidities. The preoperative characteristics of the participants are shown in Table 1. The mean operative time was 108.3 ± 21.3 min (range 80-185 min) in the LRYGB group versus 83.2 ± 23.7 min (range 60-160 min) in the LSG group, with a significant difference. The length of hospitalization and major complication rates were similar in both groups. Only one patient (in the LRYGB group) had postoperative gastrointestinal dysfunction, with severe abdominal distention and vomiting; the patient recovered after 24 days with medical treatment.

**Table 1:** Patient characteristics

Characteristics	LRYGB (*n* = 63)	LSG (*n* = 63)	*P* value
Gender			
Male	21	21	>0.99
Female	42	42	
Age (years)	33.9 ± 10.1	34.6 ± 10.4	0.70
BMI (kg/m2)	38.5 ± 5.7	38.9 ± 5.4	0.69
Waist circumference (cm)	96.1 ± 8.8	96.7 ± 8.9	0.70
Comorbidities, n (%)			
T2DM	21 (33.3)	16 (25.4)	0.33
Hypertension	16 (25.4)	17 (27.0)	0.84
Dyslipidemia	36 (57.1)	33 (52.4)	0.59
Hyperuricemia	10 (15.9)	12 (19.0)	0.64
Sleep Apnea	15 (23.8)	20 (31.7)	0.32
Operation time (min)	108.3 ± 21.3	83.2 ± 23.7	<0.001*
Hospital day (days)	5.5 ± 5.1	4.4 ± 1.2	0.10
Major complications, n (%)	1 (1.6)	0 (0)	>0.99 ^a^

LRYGB: laparoscopic Roux-en-Y gastric bypass; LSG: laparoscopic sleeve gastrectomy; BMI: body mass index; T2DM: Type 2 diabetes mellitus.

* *P* < 0.05;

a Fisher's exact test (two-sided).

**Figure 1:**
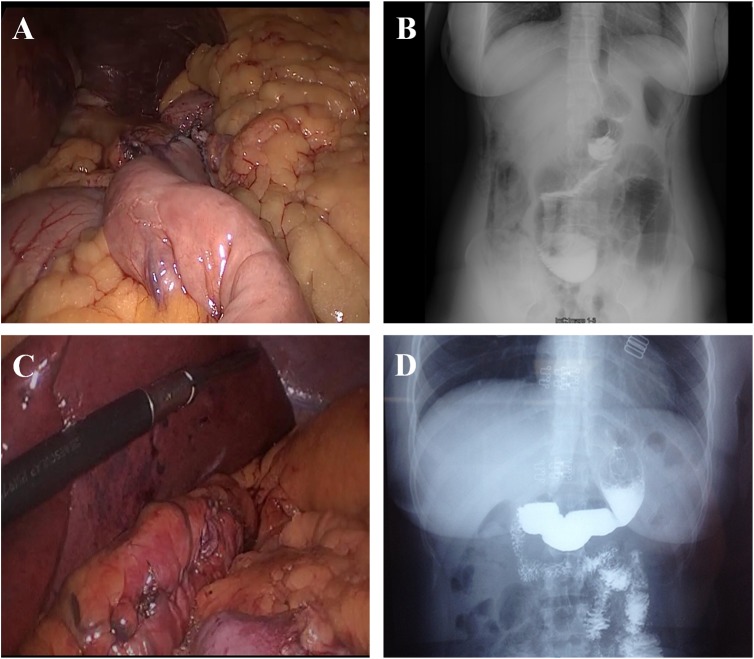
Bariatric surgery for treating obesity **A.** Intra-operative photograph of the LRYGB procedure. **B.** Postoperative upper gastrointestinal barium X-ray radiography after LRYGB. **C.** Intra-operative photograph of the LSG procedure. **D.** Postoperative upper gastrointestinal barium X-ray radiography after LSG.

**Figure 2:**
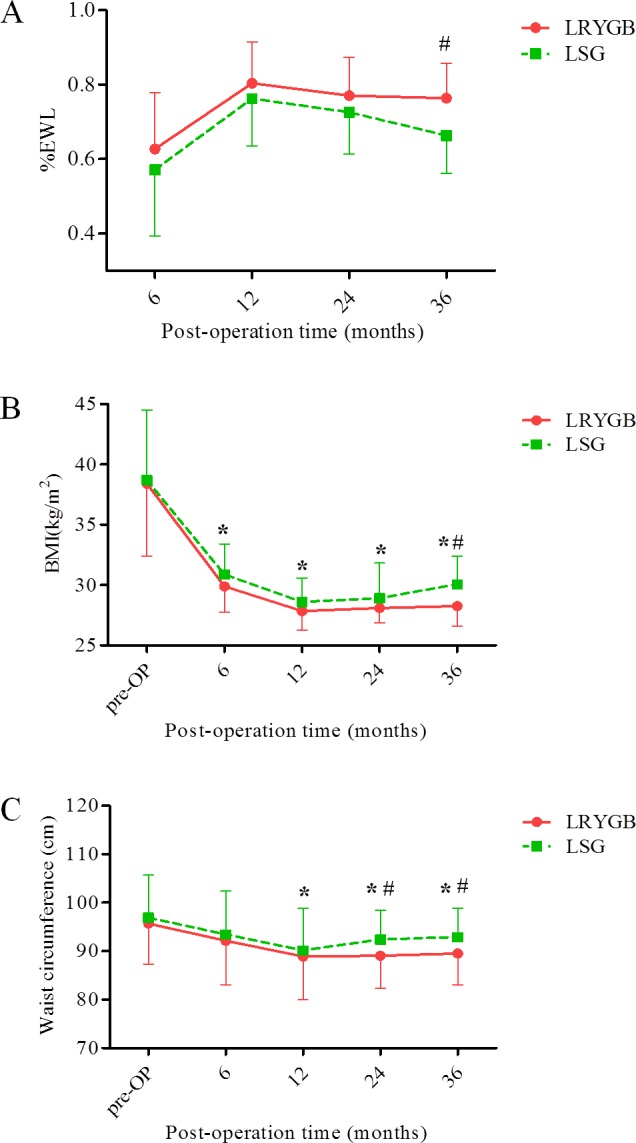
Postoperative changes of %EWL, BMI and waist circumference **A.** Mean %EWL after LRYGB and LSG (with error bars—shown above mean for LRYGB and below mean for LSG—indicating standard deviation). **B.** Mean BMI values after LRYGB and LSG (with error bars—shown below mean for LRYGB and above mean for LSG—indicating standard deviation). **C.** Mean waist circumference values after LRYGB and LSG (with error bars—shown below mean for LRYGB and above mean for LSG—indicating standard deviation). * comparison between pre- and post-operation, *P* < 0.05. ^#^ comparison between LRYGB and LSG, *P* < 0.05.

The maximum weight loss in the two groups was reached at 1 year after surgery, after that a slight weight regain appeared, but greater in the LSG group (Figure 2). After surgery, mean BMI values at all follow-up time were significantly lower than preoperative status, and waist circumference changes were also reached significant differences at 12, 24 and 36 months postoperatively. No significant differences were observed between the two groups in terms of mean %EWL at 6, 12, and 24 months (62.4 ± 14.7%, 80.1 ± 10.6%, and 77.4 ± 11.6%, respectively, in LRYGB patients *vs*. 56.3 ± 17.2%, 76.7 ± 12.9%, and 73.1 ± 10.5%, respectively, in LSG patients). The BMI in the LRYGB group was slightly, but not significantly, lower than that in the LSG group at 6, 12, and 24 months (29.8 ± 2.2, 27.9 ± 1.4, and 28.3 ± 1.1 kg/m^2^, respectively, in the LRYGB group *vs*. 30.6 ± 2.7, 28.5 ± 1.9, and 28.9 ± 3.1 kg/m^2^, respectively, in the LSG group). Similar situation could also be found in terms of waist circumference, which no significant differences occurred between two groups at 6 and 12 months (92.5 ± 9.3 and 88.7 ± 8.8 cm in the LRYGB group *vs*. 93.3 ± 9.2 and 90.8 ± 8.9 cm in the LSG group). However, at 3-year follow-up, the mean %EWL in the LRYGB group was significantly higher than that in the LSG group (76.5 ± 9.2% *vs*. 65.7 ± 10.3%, *P* < 0.05, Figure 2A), and, consequently the LRYGB group had a significantly lower BMI at 3-year follow-up (28.2 ± 1.5 kg/m^2^ *vs*. 30.9 ± 2.4 kg/m^2^, *P* < 0.05, Figure 2B). Also, the mean waist circumference values in the LRYGB group were significantly lower than that in the LSG group at 24 and 36 months follow-up (88.7 ± 6.5 and 89.8 ± 6.9 cm in the LRYGB group *vs*. 92.5 ± 6.7 and 92.7 ± 6.1 cm in the LSG group, respectively, *P* < 0.05, Figure 2C).

At follow-up 1 and 3 years after surgery, we observed good resolution or improvement of obesity-related comorbidities such as T2DM, dyslipidemia, and sleep apnea, in both groups (Tables 2 and 3), but the differences were not significant (*P* > 0.05). For T2DM, the remission rates at 1 year were 81.0% in the LRYGB group versus 68.8% in the LSG group and, at 3 years, 63.2% in the LRYGB group versus 57.1% in the LSG group.

**Table 2:** Remission of comorbidities at 1-year follow-up

Comorbidities	LRYGB		LSG		*P* value
	Preoperative	Resolution or improvement (%)	Preoperative	Resolution or improvement (%)	
T2DM	21	17 (81.0)	16	11 (68.8)	0.46 ^a^
Hypertension	16	9 (56.3)	17	7 (41.2)	0.39
Dyslipidemia	36	21 (58.3)	33	21 (63.6)	0.65
Hyperuricemia	10	4 (40.0)	12	4 (33.3)	>0.99 ^a^
Sleep apnea	15	10 (66.7)	20	12 (60.0)	0.69

LRYGB: laparoscopic Roux-en-Y gastric bypass; LSG: laparoscopic sleeve gastrectomy; T2DM: type 2 diabetes mellitus.

a Fisher's exact test (two-sided).

**Table 3:** Remission of comorbidities at 3-year follow-up

Comorbidities	LRYGB		LSG		*P* value
	Preoperative	Resolution or improvement (%)	Preoperative	Resolution or improvement (%)	
T2DM	19	12 (63.2)	14	8 (57.1)	0.73
Hypertension	14	5 (35.7)	16	4 (25.0)	0.52 ^a^
Dyslipidemia	33	17 (51.5)	32	18 (56.3)	0.70
Hyperuricemia	9	3 (33.3)	11	2 (18.2)	0.62 ^a^
Sleep apnea	13	7 (53.8)	18	8 (44.4)	0.61

LRYGB: laparoscopic Roux-en-Y gastric bypass; LSG: laparoscopic sleeve gastrectomy; T2DM: type 2 diabetes mellitus.

a Fisher's exact test (two-sided).

## DISCUSSION

LRYGB is recognized as the gold standard bariatric surgery, however, it is a technically challenging procedure, with the possibility of serious complications. LSG, which originated from biliopancreatic diversion with duodenal switch operation, was designed by Regan et al.[5] as the first step of a two-stage bariatric surgery in high-risk patients. It has proved to an effective independent procedure and has gradually gained in popularity in recent years.[6] Compared with LRYGB, LSG is less technically complex, requires less surgical time, and is less expensive.[7,8] Of the currently accepted weight loss procedures, LSG will likely become the most common bariatric operation worldwide over the next 5 years.[3] Since LSG is an emerging surgery in China, comparison with LRYGB is important to facilitate evidence-based bariatric surgery decisions. Therefore, we designed this study to compare the safety and efficiency of LSG with that of LRYGB; this kind of 1:1 paired cohort design was expected to largely eliminate other influence of factors such as preoperative BMI, gender, age, and surgery quality, and to reflect the real effect of operation itself. All patients completed follow-up for at least 1 year, which adds to the reliability of our results.

The primary endpoint of this study was the short-term and mid-term weight loss. In our series, LRYGB group had a tendency of superiority on weight loss than LSG group, and the difference reached statistical significance at 3 years post surgery. Previous studies have reported conflicting results with respect to the relative efficacy of these two procedures. We selected part of papers to compare the results from different regions of the world. The outcomes of studies from Asia, [9,10] Europe, [11] North America, [8] South America [4] and Oceania [12] are listed in Table 4. We found no studies from Africa. Mostly, the research indicated that LSG and LRYGB had similar effects on weight loss and diabetes. However, Zhang et al.[9] have reported that weight loss at 3 years after surgery was better with LRYGB, which is consistent with our finding. A recent meta-analysis that included 21 studies also showed that after 1.5 years' follow-up, LRYGB achieved a significant higher %EWL than LSG.[13] So far, therefore, there is no consensus on whether these two procedures are comparable in efficacy or whether one is superior to the other.

**Table 4:** Details of some studies comparing LRYGB and LSG from different regions

Characteristic	Our series		Zhang et al. [10]		Yang et al. [11]		Peterli et al. [12]		Lim et al. [9]		Boza et al. [4]		Thomas et al. [13]	
	LRYGB (*n*=63)	LSG (*n*=63)	LRYGB (*n*=32)	LSG (*n*=32)	LRYGB (*n*=32)	LSG (*n*=32)	LRYGB (*n*=110)	LSG (*n*=107)	LRYGB (*n*=237)	LSG (*n*=248)	LRYGB (*n*=786)	LSG (*n*=811)	LRYGB (*n*=11)	LSG (*n*=11)
Country	China		China		China		Switzerland		America		Chile		New Zealand	
Region	Asia		Asia		Asia		Europe		North America		South America		Oceania	
Publication year	-		2014		2015		2013		2014		2012		2016	
Study type	1:1 matched cohort study		RCT		RCT		RCT		Retrospective study		Case-control study		Cross-sectional study	
Study design	Patients were matched for gender, age (±8 years), and BMI (±1.5 kg/m^2^).		Computer-generated random numbers were used to allocate the type of procedure (LRYGB or LSG).		A computer generated variable block schedule was used for randomization. Allocation to treatments was not concealed.		A computer-based randomization with sealed envelopes was used to assign patients to receive either LSG or LRYGB.		The hospital database was reviewed to identify eligible patients who had undergone bariatric surgery.		Patients who underwent LSG were randomly matched by age, gender, preoperative weight, and BMI to patients undergoing LRYGB.		Patients with T2DM scheduled for either LRYGB or LSG between August 2010 and March 2012 were recruited for the study.	
Study aim	Effect on weight loss		Effect on weight loss		Effect on T2DM		Effect on weight loss and comorbidities		Effect on weight loss		Effect on weight loss and comorbidities		Effect on T2DM	
Gender (M/F)	21/42	21/42	14/18	12/20	13/19	9/23	31/79	30/77	18/219	24/224	184/602	193/618	1/10	3/8
Age (years)	33.9±10.1	34.6±10.4	32.2±9.2	29.3±9.8	41.4±9.3	40.4±9.3	42.1±11.2	43.0±11.1	54(M), 40(F)	52(M), 39(F)	37.0±10.3	36.4±11.7	41	45
BMI (kg/m^2^)	38.5 ± 5.7	38.9 ± 5.4	39.3±3.8	38.5±4.2	32.3±2.4	31.8±3.0	44.2±5.3	43.6±5.3	41(M), 41(F)	42(M), 40(F)	38.0±3.4	37.9±4.6	44.5	42.2
Major complications (n)	1	0	5	1	0	0	11	2*	-	-	152 (all complicaitons)	51*(all complicaitons)	-	-
%EWL at 6 m	62.4	56.3	-	-	74.9	67.3*	-	-	-	-	84.6	80.5	-	-
%EWL at 1 y	80.1	76.7	84.5	73.9	86.4	79.6*	-	-	72	64.7*	97.2	86.4*	-	-
%EWL at 3 y	76.5	65.7*	79.8	68*	92.0	81.93*	72.8	63.3	-	-	93.1	86.8	-	-
%EWL at 5 y	-	-	76.2	63.2*	-	-	-	-	68.3	57.4	-	-	-	-
HbA1c (%) at 1 y	-	-	-	-	5.8	5.9	-	-	-	-	5.9	5.7	-	-
HbA1c (%) at 3 y	-	-	-	-	5.7	5.9	-	-	-	-	-	-	-	-
T2DM R/I rate (%) at 1 y	81.0	68.8	-	-	-	-	67.9	57.7	-	-	-	-	-	-
T2DM R/I rate (%) at 3 y	63.2	57.1	-	-	92.6	89.3	-	-	-	-	93.2	100	-	-
T2DM R/I rate (%) at 5 y	-	-	87.5	88.9	-	-	-	-	-	-	-	-	-	-
Conclusion	LSG is inferior to LRYGB in mid-term weight loss, but similar in safety and improvement of comorbidities.		LRYGB is superior in terms of weight loss.		LRYGB and LSG have similar effect on diabetes.		LRYGB and LSG are almost equally efficient in achieving weight loss and improvement of comorbidities.		LRYGB and LSG have similar effect on long-term weight loss		LRYGB and LSG result in similar weight loss and remission of comorbidities.		LRYGB and LSG improve glucose metabolism through different effects on pancreatic beta-cell function, insulin sensitivity, and free fatty acids.	

LRYGB: Laparoscopic Roux-en-Y gastric bypass; LSG: laparoscopic sleeve gastrectomy; BMI: body mass index; T2DM: type 2 diabetes mellitus; %EWL: excess weight loss percentage; HbA1c: glycated hemoglobin; R/I rate: resolution or improvement rate.

* compared with LRYGB, *P* < 0.05.

If the difference of weight loss effect between two procedures real exists, as our result and some authors demonstrated, that could be couple of reasons for this. First, LRYGB is a hybrid procedure, reducing stomach capacity as well as absorption of nutrition. But LSG, as a partial gastrectomy, is only a restrictive procedure and does not offer any known mal-absorptive characteristics. Second, some important hormones are known to play key roles in weight loss and the remission of comborbidities. For the LRYGB, evidences showed some anorectic hormones such as glucagon-like peptide-1 (GLP-1) and peptide YY increased significantly post surgery.[14] Furthermore, studies have demonstrated that LRYGB might promote weight loss by reducing food cues in mesolimbic pathways.[15] With regard to LSG, researchers have found marked postoperative decrease in fasting and postprandial levels of ghrelin, which is an important hormone associated with weight loss. But this decrease in secretion has not been observed following LRYGB.[16] Therefore, the mechanisms whereby these two procedures bring about weight loss might be totally different. Third, as some studies including our revealed, LSG may be as effective as LRYGB for weight loss over the short term, but inferior for mid-term or long-term weight loss. As LSG is only a restrictive procedure (without influence on absorption), poor postoperative compliance with diet control may lead to in gradual expansion of the sleeved stomach and offset the early benefits of surgery. Last, differences in study designs can also contribute to the discrepancies seen in the literature. A prospective, multicenter, randomized clinical trial with long-term follow-up is necessary for elucidating the differences between LRYGB and LSG.

A secondary goal of this study was to assess the resolution or improvement of obesity-associated comorbidities following surgery. We found noteworthy rates of resolution or improvement of comorbidities in both groups, confirming the beneficial metabolic effect of both LSG and LRYGB. There was no significant difference between the groups in the remission rates of comorbidities at 1 year and 3 years in our series; other authors have reported the similar outcomes (Table 4).[4, 10, 11] A recent meta-analysis of 62 studies, however, has indicated that while LSG is equivalent to LRYBD with regard to improvement in T2DM and sleep apnea, it is inferior to LRYGB for remission of hypertension, dyslipidemia, gastroesophageal reflux disease, and arthritis.[17] Thus, although the remission rates of comorbidities were generally satisfactory, we found large variations in the results from different cohorts and from different countries. Differences in the indications for surgery as well as variations in sample sizes and study designs might be responsible for the disparity.

Data on the safety of the two procedures also presents wide divergence. In our series, operation time was significantly shorter in the LSG group. We found no significant difference in major complications between two procedures, which is consistent with the results from Zhang et al. and Kehagias et al. [9,18] However, Table 4 also showed that in the studies from Switzerland and New Zealand, complications were significantly higher in the LRYGB group, which was contrary to a recent meta-analysis's finding.[19] We did not observe any leakage or bleeding in our series; routinely utilizing suture reinforcement and small sample size in this study might be possible reasons for this good result.

## LIMITATIONS

Our study had several limitations. First, it was a retrospective cohort study from a single center, with a relatively small number of patients due to the restrictive matching criterion. Second, the surgical indications of this study were based on the eastern guidelines, which are different from the West such as NIH criteria in 1991. The starting and average BMI values of patients were lower than the western reports, which would obviously affect the long term %EWL in favor of a higher percentage. Therefore, the outcome of this study might be only suitable for Eastern or this Chinese population. Third, the loss to follow-up, a common problem in cohort studies, was > 5% at 3 years after surgery, although less than 10%; and this would undoubtedly affect our mid-term outcome assessment. Last but not least, there is always the possibility that our results were confounded by some unknown variables, such as patient compliance with postoperative advice, especially with regard to diet control and lifestyle changes.

## CONCLUSIONS

In conclusion, both LRYGB and LSG are safe and effective bariatric procedures, with similar complication rates and improvement of comorbidities. However, LRYGB seems to be superior to LSG with regard to mid-term weight loss. Multicenter prospective researches with longer follow-up are required to further elucidate the long-term efficacy and safety of the two procedures.

## MATERIALS AND METHODS

### Patients

This retrospective study was conducted of patients who received LRYGB or LSG between January 1, 2009, and January 31, 2013, in West China Hospital, Sichuan University in China. All human studies were performed in accordance with the principles of the Declaration of Helsinki. The present study was approved by the Research and Ethics Committee of West China Hospital, and informed consent was obtained from all patients.

In this study, basic inclusion criteria for bariatric surgery were according to the guideline of the Chinese Society for Metabolic and Bariatric Surgery (CSMBS), which were an age of 18 to 60 years, BMI ≥ 32 kg/m^2^ or BMI ≥ 27.5 kg/m^2^ with one or more comorbidities, such as type 2 diabetes mellitus (T2DM), dyslipidemia, and hypertension. Additionally, all patients selected for surgery were those who had failed to achieve weight loss or resolution of comorbidities with lifestyle changes and medication, or symptom recurred after such treatments. The criteria for diagnosis of comorbidities were as reported previously.[20] Patients were excluded from the study if they had previous gastric cancer surgery or history of severe systemic or mental disease. Patients were retrospectively selected from the Bariatric and Metabolic Surgery Database of West China hospital. The restricted 1:1 matching criteria were same gender, age ± 8 years and BMI ± 1.5 kg/m^2^. Prior to surgery, all patients underwent a multidisciplinary evaluation by internists, psychiatrists, and surgeons. All the procedures in both groups were performed by the same surgeon (Cheng Z), deputy chairman of CSMBS, who has performed more than 400 laparoscopic bariatric surgeries.

Before surgery, we recorded age, gender, education, height, weight, BMI, waist circumference, blood pressure, and details regarding obesity-related comorbidities. The operative time, postoperative complications, and length of hospitalization were also recorded.

### Surgical techniques

All procedures were performed under general anesthesia. LRYGB was conducted with a 100-cm biliopancreatic limb and a 100-cm alimentary limb. A 45-mm endoscopic stapler with 3.5-mm staple height was used for creating a gastric pouch with around 20 mL capacity. The gastrojejunostomy and the jejunojejunostomy were performed using a linear laparoscopic stapler, with staple heights of 3.5-mm and 2.5-mm, respectively. The mesenteric defects were closed in all cases.

LSG was performed laparoscopically using the 4-trocar technique. The gastric dissection began at 5 cm from the pylorus of stomach. Then the dissection was continued to go along with the greater curvature to the angle of His, using the linear laparoscopic stapler. The size of the sleeve was controlled using a 34-Fr bougie on each case. The staple line was routinely oversewn with absorbable running suture. At the end of the procedure, leakage and bleeding were checked for. The intra-operative photographs and postoperative upper gastrointestinal barium X-ray radiographies are presented in Figure 1.

### Follow-up

Patients were requested to attend follow-up at 6, 12, 24, and 36 months after surgery. All patients completed at least 1 year of follow-up. However, 2 LRYGB patient and 1 LSG patient failed to attend follow-up at the end of 2 years; at the end of 3 years, an additional 2 LRYGB patients and 2 LSG patients were lost to follow-up.

At each follow-up visit, the percentage of excess weight loss (%EWL), current BMI and waist circumference, comorbidities, and complications were recorded. Any condition necessitating re-hospitalization and medical or surgical intervention was regarded as a major complication. %EWL was the major measure index for the effect of weight loss, the procedure was considered inadequate if the %EWL was < 50% but >30%, and a failure if %EWL < 30% at 1-year post operation. The criteria for remission or improvement of comorbidities were as follows. With respect to T2DM, remission was defined as fasting blood glucose (FPG) < 5.6 mmol/L and glycated hemoglobin (HbA1c) < 6% under no medication, and improvement was regarded as using lower doses of medication, or reduction of FPG > 1.39 mmol/L or reduction of HbA1c > 1%. Remission of hypertension was described as blood pressure was below 120/80 mm Hg under no medication, and improvement was regarded as any reduction in the hypertension medication. Remission of hyperlipidemia and hyperuricemia were defined as cholesterol, triglyceride and uric acid were below the cut-off point under no medication, any reduction in the medication was considered as improvement. Symptoms of sleep apnea were diagnosed as repeated upper airway occlusions and the need for continuous positive airway pressure during sleep with or without sleepiness. Remission of symptoms was established when breathing pauses during sleep were no longer experienced. Obvious reduction of episode times was considered as improvement.

### Statistical analysis

Continuous variables were expressed as means ± standard deviation (SD). The independent samples *t* test was used to compare continuous variables, and either the Chi-square test or Fisher's exact test (two-sided) was used for categorical variables. SPSS 17.0 (SPSS Inc., Chicago, IL, USA) was employed for all analyses. GraphPad Prism 6.0 (GraphPad Software Inc., San Deigo, CA, USA) was used for generating the graphics. Statistical significance was set at *P* < 0.05.
